# Safety and Efficacy of Caproamin Fides and Tranexamic Acid Versus Placebo in Patients Undergoing Coronary Artery Revascularization

**DOI:** 10.15171/jcvtr.2014.011

**Published:** 2014-09-30

**Authors:** Alireza Alizadeh Ghavidel, Ziae Totonchi, Mitra Chitsazan, Maziar Gholampour Dehaki, Farshid Jalili, Fariborz Farsad, Maral Hejrati

**Affiliations:** ^1^Heart Valve Disease Research Center, Rajaei Cardiovascular Medical and Research Center, Iran University of Medical Science, Tehran, Iran; ^2^Rajaei Cardiovascular Medical and Research Center, Iran University of Medical Science, Tehran, Iran; ^3^Rasoul-e-Akram General Hospital, Iran University of Medical Sciences, Tehran, Iran

**Keywords:** Aminocaproic acid, Antifibrinolytic agents, Coronary artery bypass, Tranexamic acid

## Abstract

*Introduction:* Excessive fibrinolysis contributes to post-cardiopulmonary bypass bleeding. Tranexamic Acid (TXA) and Caproamin Fides are synthetic lysine analogues that inhibit plasminogen-fibrin binding. The present study aimed to compare TXA and Caproamin Fides versus placebo in patients undergoing elective coronary artery revascularization.

*Methods:* We analyzed perioperative data of 300 adult patients undergoing coronary artery revascularization. Patients were randomly allocated to receive TXA (n=100), Caproamin Fides (n=100) or placebo (n=100) during perioperative time. Mediastinal bleeding during the first 24 hours post-operation, transfusion requirement and post-surgical complications were assessed.

*Results:* Most descriptive and intra-operative parameters were well comparable between the 3 study groups. Except for mean number of packed red blood cell (PRBC) units transfused during ICU stay (P=0.01), patients in the Caproamin Fides and TXA groups did not show any statistically significant differences regarding transfusion of blood products during peri-operative period. There was no evidence of a significant difference in mediastinal blood loss during the first 24 hours post-operation between the patients receiving TXA or placebo, while patients in the Caproamin Fides group had significantly lower mediastinal bleeding than the other 2 groups (Caproamin Fides vs. placebo, P=0.002, <0.001 and <0.001 at 6, 12 and 24 hours post-operation; Caproamin Fides vs. TXA, P=0.009, 0.003, <0.001 at 6, 12 and 24 hours post-operation). The incidence of postoperative complications were comparable between Caproamin Fides and TXA groups (P>0.05).

*Conclusion:* In conclusion, Caproamin Fides seems to be superior to TXA regarding the blood saving effects in patients undergoing coronary artery revascularization.

## Introduction

Postoperative bleeding threats patients undergoing cardiac surgeries. Using blood products in cardiac surgeries was estimated over 10-15% in England, and 20% in United States.^1^ Re-exploration due to continued bleeding is needed in 3-5% of the cases.^2^ Blood loss and transfusion of blood products increase the morbidity and mortality. This is accompanied with transfusion related complications such as febrile non hemolytic reactions, ABO blood group mismatch, anaphylactic reactions and infection transmission. Excessive fibrinolysis and transient platelet function impairment are assumed as the causes of post-cardiopulmonary bypass bleeding.^3^ Thus, it may be sensible that anti-fibrinolytic agents [such as aprotinin, tranexamic acid (TXA), ɛ-aminocaproic acid] can reduce blood loss.^4^ Aprotinin was stopped early due to renal dysfunction, myocardial infarction and sudden death followed by its administration.^5,6^ TXA and ɛ-aminocaproic acid (Caproamin Fides^®^) are now routinely used in cardiac surgeries. These are synthetic lysine analogues that inhibit plasminogen-fibrin binding. It has been reported that TXA is 10 times more potent than Caproamin Fides in reducing postoperative blood loss.^7,8^ However, there is not general consensus regarding the most appropriate agent that can be used in coronary artery bypass grafting (CABG). As a result, the present study was designed to compare Caproamin Fides and TXA versus placebo in a group of patients undergoing elective coronary artery revascularization.

## Materials and methods

The study after approval by the Research Ethics Committee of Iran University of Medical Sciences was conducted at the Rajaei Cardiovascular, Medical and Research Center between October 2010 and November 2012. Three hundred patients gave informed consent to participate in this prospective, randomized, double-blind, placebo-controlled trial. Exclusion criteria included a serum creatinine level of >2 mg/dl, previous history of bleeding or coagulation disorders, taking oral anticoagulation medications within 72 hours of the surgery and allergy to the study medications. Eligible patients older than 18 years scheduled to undergo non-emergent CABG were randomly allocated, via a computer-generated random number list, to receive one of the three study regimens, i.e., TXA (n=100), ɛ-aminocaproic acid (Caproamin Fides^®^, n=100) or the placebo (n=100). Under general anesthesia cardiopulmonary bypass (CPB) machine was established after heparin (3 mg/kg) administration. Additional dose of heparin was used during CPB to maintain the activated coagulation time higher than 380 seconds. Blood flow rate of 2.4 L.min^-1^.m^2^ were obtained with a roller pump. Mild systemic hypothermia (between 32 °C and 34 °C) was maintained during aortic cross-clamping. For TXA group, the primary dose of 10 mg/kg via prime solution and the maintenance dose of 0.5-2 mg/kg/h in proportion to serum creatinine were given. For Caproamin Fides group 150 mg/kg was given via prime solution and 1 gr/h during operation. The medications were discontinued on separation from CPB. The control group received the placebo solution in a similar way to the study medications. Placebo consisted of 0.9% normal saline solution. Normal saline and study medications were prepared in equivalent volume in 50 ml syringes with a coded label by a nurse who was not included in the study.

After separation from CPB, the effects of heparin were reversed with protamine sulfate (3 mg/kg or a dose equivalent to heparin dose) to decrease activated clotting time to 120 seconds. Additional doses of protamine were used if the target activated clotting time was not achieved. Blood remaining in the CPB circuit after separation was collected and infused to the patient. The mediastinal drainage fluid was not infused to the patients. Packed red blood cells (PRBCs) were administered to maintain hematocrit around 19-22% during CPB and hemoglobin concentration of at least 8 g/dl after CPB as long as patients are hemodynamically stable.

During post-operative period fresh frozen plasma (FFP) was administered as needed to keep activated partial thromboplastin time (aPTT) between 50-70 seconds and platelet was used to keep platelet count above 80,000 in all patients. Hemoglobin concentration was kept above 8 g/dl in hemodynamically stable patients and above 10 g/dl in patients with debilitating chronic diseases such as chronic heart failure or chronic obstructive pulmonary disease.

Clinical data compared between groups included basic demographic data; duration of CPB, aortic cross-clamping, and operation; and postoperative surgical and medical outcomes. Blood loss during the operation was not evaluated. The total volume of mediastinal bleeding during the first 24 hours after surgery was measured hourly by the ICU nurses. Transfusions of PRBCs and hemostatic blood products (platelets, FFP) during the operation, ICU stay and the surgery ward admission were recorded. Laboratory data collected for each patient included the platelet count and routine coagulation profile (thrombin time, prothrombin time, and activated partial thromboplastin time) which were measured prior to induction of anesthesia and on arrival in the ICU. Hematocrit concentrations at the time of discharge from the hospital were compared with preoperative values. Serum creatinine concentrations were measured the day before operation and on the first postoperative day.

### Statistical analysis

All analyses were conducted by Statistical Package for Social Sciences (SPSS) software, version 19 (SPSS, Chicago, IL). Normality of all data was initially assessed using the Kolmogorov-Smirnov test. Quantitative variables were presented as means ± standard deviation (SD) for normally distributed variables and as median (interquartile range, IQR) for variables without normal distribution. Categorical data were presented as numbers and percentages. Categorical data were compared by the chi-square test whereas quantitative ones were compared by the Student’s t-test, the Manne-Whitney test, the Kruskale Wallis test, One-Way ANOVA and repeated measurement ANOVA as appropriate. Relationships were assessed using Pearson, Spearman or Kendall tests as appropriate. All P-values were two-tailed and P<0.05 was considered statistically significant.

## Results

### Patient characteristics

Three hundred patients including 210 (70%) male and 90 (30%) female were enrolled in the study. The mean age was 45.25±15.53 years. Comparisons of perioperative characteristics of the patient groups are presented in Table 1.

**Table 1:** Perioperative characteristics of the patients

	**Caproamin Fides** ** (n=100)**	**TXA** **(n=100)**	**Control** **(n=100)**	**P**
Male	75 (75)	70 (70)	65 (65)	0.30
Age	59±10	58±9	59±10	0.83
BSA (kg/m^ 2 ^)	1.88±0.14	1.77±0.14	1.78±0.18	<0.001*
Hemoglobin (g/dL)	14.32±1.71	13.97±2.02	14.21±1.54	0.65
Diabetes	42 (42)	39 (39)	51 (51)	0.20
HTN	40 (40)	40 (40)	47 (47)	0.51
History of MI	30 (30)	29 (29)	40 (40)	0.18
Renal failure	1 (1)	7 (7)	13 (13)	0.004*
Duration of taking clopidogrel (months)	48	44	36	0.21
Frequency of surgery				0.44
First surgery	98 (98)	99 (99)	96 (96)	
Redo surgery	2 (2)	1 (1)	4 (4)	
Graft no.	3.29±0.90	3.50±0.75	3.37±0.77	0.76
AOX time (min)	41±19	46±19	44±21	0.20
CPB time (min)	76±28	86±28	90±40	0.01*
Operation time (min)	260±50	241±54	241±63	0.02*

Data are shown as number (percentage) and mean±standard deviation. AOX= aortic cross-clamping; BSA= body surface area; CPB= cardiopulmonary bypass; HTN= hypertension; MI= myocardial infarction. *Statistically significant.

### Blood products transfusion

Sixty five (65%) patients in Caproamin Fides group, 70 (70%) patients in TXA group and 79 (79%) patients in the control group received 1-6 units of packed cell intraoperatively (P=0.04). The mean number of units of transfused packed cell were 1.13±1.09, 1.30±1.08 and 1.68±1.37 units in Caproamin Fides, TXA and the control groups (P=0.004), respectively.

During ICU stay, 53 (53%) patients in Caproamin Fides group received an average of 0.78±0.60 packed cell units, 60 (60%) patients in TXA group received mean units of 1.25±1.01 packed cell and 74 (74%) patients in the control group were transfused mean units of 1.65±1.15 packed cell (P<0.001). The percentage of patients receiving blood product transfusions and the number of units of blood products administered to the patients are shown in Table 2.

**Table 2:** Allogeneic blood product transfusions.

		**Caproamin Fides** **(n=100)**	**TXA** **(n=100)**	**Control** **(n=100)**	**Caproamin Fides** **vs. control**		**TXA vs. control**		**Caproamin Fides** **vs. TXA**	
					**Effect size**	**P**	**Effect size**	**P**	**Effect size**	**P**
Transfusions During Operation	PRBCs (pts)	65 (65)	70 (70)	79 (79)	RR=0.49 (0.26-0.93)	0.02*	RR=0.62 (0.32-1.18)	0.14	RR=1.25 (0.69-2.27)	0.45
	FFP (pts)	5 (5)	7 (7)	11 (11)	RR=0.42 (0.14-1.27)	0.12	RR=0.66 (0.22-1.64)	0.32	RR=1.43 (0.43-4.66)	0.55
	Platelets (pts)	4 (4)	5 (5)	6 (6)	RR=0.65 (0.17-2.38)	0.51	RR=0.82 (0.24-2.79)	0.75	RR=1.26 (0.32-4.84)	0.73
	PRBCs (U)	1.13±1.09	1.30±1.08	1.68±1.37	MD=-0.55	0.001*	MD=-0.38	0.02*	MD=-0.17	0.31
	FFP (U)	0.22±0.99	0.24±0.92	0.45±0.99	MD=-0.23	0.14	MD=-0.21	0.18	MD=-0.02	0.89
	Platelets (U)	0.13±0.79	0.19±0.89	0.22±0.91	MD=-0.07	0.57	MD=-0.03	0.80	MD=-0.04	0.74
Transfusions During ICU Stay	PRBCs (pts)	53 (53)	60 (60)	74 (74)	RR=0.31 (0.17-0.56)	0.001*	RR=0.52 (0.28-0.96)	0.03*	R=1.69 (0.96-2.96)	0.06
	FFP (pts)	19 (19)	23 (23)	38 (38)	RR=0.38 (0.20-0.72)	0.003*	RR=0.48 (0.26-0.90)	0.02*	R=1.27 (0.64-2.52)	0.48
	Platelets (pts)	5 (5)	6 (6)	8 (8)	RR=0.60 (0.19-1.91)	0.39	RR=0.73 (0.24-2.19)	0.58	R=1.21 (0.35-4.11)	0.75
	PRBCs (U)	0.78±0.08	1.25±0.53	1.65±0.55	MD=-0.87	<0.001*	MD=-0.40	0.04*	MD=-0.47	0.01*
	FFP (U)	0.57±0.28	0.84±0.69	1.30±0.92	MD=-0.73	0.002*	MD=-46	0.05*	MD=-0.27	0.24
	Platelets (U)	0.22±0.01	0.30±0.20	0.39±0.15	MD=-0.17	0.33	MD=-0.09	0.61	0.08	0.65

Data are shown as number (percentage) and mean±standard deviation. FFP= fresh frozen plasma; MD= mean difference; PRBCs= packed red blood cells; pts= patients; RR= relative risk; U= units. * Statistically significant.

After discharge from ICU and during the hospitalization in the surgery ward PRBC was transfused in 1% of patients in Caproamin Fides group, 2% in TXA group and 8% in the control group (P<0.001).

### Postoperative bleeding

**Figure 1:**
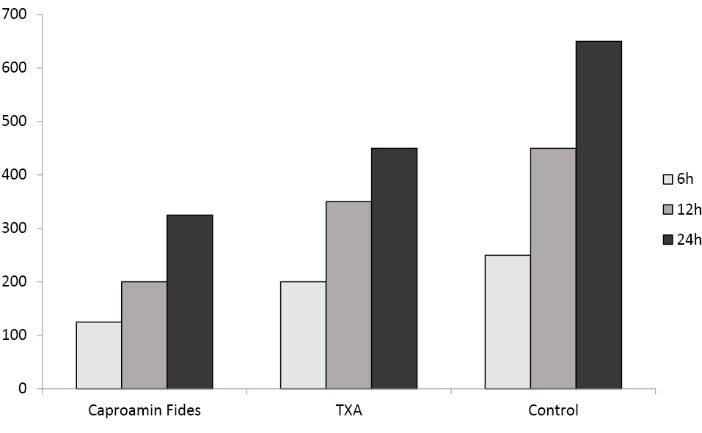
Post-operative mediastinal bleeding (mL) in the 3 study groups during the first 24 hours.

Comparison of the post-operative mediastinal bleeding in the first 24 hours between the 3 study groups is shown in Table 3. The median volumes of chest drainage during the first 6 hours after surgery were 125 mL (IQR: 50-250), 200 mL (IQR: 100-400) and 250 mL (IQR: 100-438) in Caproamin Fides, TXA and the control groups, respectively (P=0.002). The median volumes of chest drainage during the first 12 hours after surgery were 200 mL (IQR: 150-400), 350 mL (IQR: 200-600) and 450 mL (IQR: 250-638) in Caproamin Fides, TXA and the control groups, respectively (P<0.001). The median volumes of total chest drainage during the first 24 hours postoperation were 325 mL (interquartile 200-550), 450 mL (interquartile range: 312.5-800) and 650 mL (IQR: 350-869) ml in Caproamin Fides, TXA and the control groups, respectively (P<0.001). Figure 1 shows post-operative mediastinal bleeding in the 3 study groups during the first 24 hours.

**Table 3:** Post-operative mediastinal bleeding in the 3 study groups.

**Mediastinal Bleeding (mL)**	**Caproamin Fides (n=100)**	**TXA** **(n=100)**	**Control** **(n=100)**	**P**	**Caproamin Fides vs. control**	**TXA vs. control**	**Caproamin Fides** **vs. TXA**
					**P**	**P**	**P**
6 hours	125 (50-250)	200 (100-400)	250(100-437.5)	0.002*	0.001*	0.29	0.009*
12 hours	200 (150-400)	350(200-600)	450(250-637.5)	<0.001*	<0.001*	0.10	0.003*
24 hours	325 (200-550)	450(312-800)	650(350-868.75)	<0.001*	<0.001*	0.10	<0.001*

Data are shown as the median (interquartile range). *Statistically significant.

Our data demonstrated that Caproamin Fides was associated with a mean reduction of around 107 ml per patient in the volume of chest drainage during the first 6 hours after surgery (P=0.002), as well as mean reductions of 173 and 240 ml per patient in the postoperative chest drainage during first 12 and 24 hours as compared to the control group; respectively (both P<0.001).

On the other hand, TXA reduced postoperative drainage during the first 6 hours on an average of 25 ml per patient (P=0. 46), and during the first 12 and 24 hours post-operation on averages by 66 ml (P=0. 15) and 95 ml (P=0. 07) per patient, respectively; as compared to the placebo group.

We also find that mean volume differences of post-operative bleeding between Caproamin Fides and TXA were 82, 107, 146 ml per patient during the first 6, 12 and 24 hours post-operation (P=0.01, 0.02 and 0.006 respectively); implying the higher efficacy of Caproamin Fides in the reduction of post-operative drainage.

According to our results, during ICU stay Caproamin Fides decreased the need for packed cell transfusion as compared to the TXA and control groups (P=0.01 and <0.001, respectively). Moreover, patients receiving TXA had lower need to packed cell transfusion comparing control group (P=0.04). Similarly, Caproamin Fides had comparable effects on FFP transfusion (P=0.24), although the effect of Caproamin Fides was more prominent as compared to control group (P=002). On the other hand, platelet productions were transfused in 5% of patients in the Caproamin Fides group, in 6% in TXA and in 8% in control groups during the ICU stay, which shows no statistically significant differences in this regard between the 3 groups (P=0.67).

Our results also showed that after discharge of ICU and during the hospitalization in the surgery ward packed cell was transfused in 1% of patients in Caproamin Fides group, 2% in TXA group and 8% in the control group (P<0.001).

Although blood loss during CABG and after it decreased mean levels of hematocrit by 5.63%, 5.81% and 5.93% in Caproamin Fides, TXA and control groups; respectively (all P<0.001), the mean decline in the hematocrits from their baseline levels were comparable in all 3 groups (P=0.46).

### Postoperative outcomes

Incidences of pericardial effusion which occurred in 1%, 4% and 3% of patients in Caproamin Fides, TXA and control groups, respectively; did not differ statistically significantly between the 3 groups (P=0.65). However, patients in Caproamin Fides group had the lowest incidence of pleural effusion (16%), followed by control (22%) and TXA (31%) groups (P=0.40). The differences in the incidence of post-op MI were inconspicuous between all the 3 groups (4%, 8% and 6% in Caproamin Fides, TXA and control groups, respectively; P=0.49). Though electrocardiographic changes seldom occurred in 11% of patients receiving TXA, and in 2% and in 5% of patients in Caproamin Fides and control groups, respectively (P=0.02). Six cases (6%) were complicated with tamponade which underwent pericardiotomy with a subxiphoid approach. Thirteen (4.3%) patients re-explored due to mediastinal bleeding and in 10 (3.3%) of these patients a surgical cause was found. Six patients (2%) complicated by superficial wound infection and 4 (1.3%) by deep wound infection. The incidences of other postoperative complications based on the three study groups are shown in Table 4.

**Table 4:** Incidences of postoperative complications

**Complication**	**Caproamin Fides** ** (n=100)**	**TXA** **(n=100)**	**Control** **(n=100)**	**P**
Cardiac tamponade	1 (1)	3 (3)	2 (2)	0.60
Clinical MI	4 (4)	8 (8)	6 (6)	0.49
Infection				0.26
Superficial	0	3 (3)	3 (3)	
Deep	1 (1)	1 (1)	2 (2)	
Pleural effusion				0.40
Chest tube	1 (1)	2 (2)	2 (2)	
Follow up	0	2 (2)	1 (1)	
Pericardial effusion	16 (16)	22 (22)	31 (31)	0.04*
Bleeding				0.44
Surgical	3 (3)	4 (4)	3 (3)	
Non-surgical	0	1 (1)	2 (2)	
Other				0.15
Neurologic	0	1 (1)	1 (1)	
Cardiac	0	2 (2)	1 (1)	
Renal	0	0	1 (1)	
Pressure sore	0	0	1 (1)	

Data are shown as number (percentage) *Statistically significant.

The duration of ICU stay was not statistically different among the 3 groups (P=0.31), although patients in Caproamin Fides had shorter duration of hospitalization as compared to the other 2 groups (P=0.03).

## Discussion

In the present study we compared the effects of TXA and Caproamin Fides over placebo in patients undergoing coronary artery bypass graft surgery. We assessed the differences between the two medications regarding post-operative bleeding and major clinical outcomes.

The main finding of the current investigation is that Caproamin Fides seemed to be superior over TXA in reducing blood loss after CABG and subsequently the need for packed cell transfusion, although both medications had comparable effects on the measured outcomes such as renal and cardiovascular complications.

In the present study, we did not measure the volume of blood loss during surgery. However, we assumed that the frequency of packed cell transfusion during surgery can indirectly reflect the extent of intraoperative bleeding. Our data showed that although both TXA and Caproamin Fides reduced the need for packed cell transfusion during surgery, patients in Caproamin Fides group received lower units of packed cell intraoperatively as compared to TXA group; though no significant difference was seen in the need for fresh frozen plasma (FFP) and platelet transfusion in all the three groups. However, Henry et al (9) in a systematic meta-analysis found that TXA in patients undergoing cardiac surgery was associated with an average volume of 287 ml per patient reduction in intraoperative bleeding comparing to control [3 trials; weighted mean difference (WMD)= -287.16 ml, 95% CI -481.57 to -92.75 ml] while the mean volume of blood loss in patients receiving ɛ-aminocaproic acid was 214 ml per patient (2 trials; WMD -213.58 ml, 95% CI -310.03 to -117.13 ml). Moreover, Raghunathan et al. showed that the use of FFP in patients receiving TXA is 17% less than in those receiving ɛ-aminocaproic acid (10).

Falana et al. found no significant differences in the postoperative blood loss, rate of re-operation for bleeding and transfusion of blood products between TXA and ɛ-aminocaproic acid in 120 patients undergoing cardiovascular surgery with or without CPB (11). They also demonstrated a comparable incidence of thromboembolic events, postoperative renal dysfunction, seizure, and 30-day all-cause mortality between these two antifibrinolytics. Given the considerable cost difference and comparable efficacy and safety, they suggest ɛ-aminocaproic acid to be used for reducing cardiovascular surgical bleeding. Makhija et al. also compared the efficacy and safety of TXA to ɛ-aminocaproic acid in 64 adult patients undergoing thoracic aortic surgery with CPB (12). They showed that cumulated mean blood loss, total PRBCs and blood product requirement up to 24 hours postoperatively were comparable between groups. However, their results revealed significant renal injury and increased tendency for renal failure in ɛ-aminocaproic acid and an increased tendency of seizure with TXA. In another study, Martin et al. also showed comparable effects of TXA and ɛ-aminocaproic acid on perioperative blood loss, rate of re-operation for bleeding and transfusion of blood products in 234 pediatric patients undergoing cardiac surgery (13).

Our data revealed that neither administration of antifibrinolytic nor the type of antifibrinolytic (TXA versus Caproamin Fides) could positively affect the extent of the hematocrit decrease in our study population after CABG surgery.

We assumed that pericardial and pleural effusions can indirectly reflect post-operative hemorrhagic complications. In this regard, our results showed that incidences of pericardial effusion which occurred in 1%, 4% and 3% of patients in Caproamin Fides, TXA and control groups, respectively; did not differ statistically significantly between the 3 groups. However, the results represent that patients in Caproamin Fides group had the lowest incidence of pleural effusion (16%), followed by control (22%) and TXA (31%) groups.

Inhibition of fibrinolysis after administration of anti-fibrinolytics results in a hypercoagulable state and concerns might arise regarding the increase of acute graft thrombosis in patients undergoing CABG. To answer this question, we compared the incidences of post-operative myocardial infarction in the study groups. Myocardial infarction was diagnosed when 2 of the following criteria were met: significant ST-T changes, an increase in the cardiac biomarkers [Creatine phosphokinase-MB (CPK-MB) and cardiac Troponin I], and new myocardial wall motion abnormality seen on echocardiography. Based on our results, the differences in the incidence of post-op MI, which might be due to graft thrombosis, were not statistically significant between all the 3 groups. However, the incidence of post-op MI was twice in TXA group as compared to Caproamin Fides group. Hence, it seems the Caproamin Fides is more effective and safe. Further study with larger number of patients could answer to this question. However, angiography was not performed in these patients to confirm the graft thrombosis as the etiology of recent infarction.

Furthermore, comparing post-operative clinical outcomes including cerebral thromboembolic events, pulmonary, renal and infective complications showed that all the 3 groups were statistically comparable. No cases of deep vein thrombosis were seen. Anti-fibrinolytic agents (TXA, ɛ-aminocaproic acid) also have not show obvious correlation with renal failure or thrombosis in a study by Tzortzopoulou et al (14). Martin et al also found statistically similar incidence of postoperative complications such as seizures and other neurological complications, renal injury, renal failure, vascular thrombosis and the in-hospital mortality in both group of pediatric patients receiving TXA or ɛ-aminocaproic acid undergoing cardiac surgeries (13).

Moreover, our results demonstrated that patients in Caproamin Fides group had shorter duration of hospitalization as compared to the other 2 groups; although all 3 groups had comparable duration of ICU stay.

## Conclusion

In conclusion, the results of our study indicate that Caproamin Fides seems to be superior to TXA regarding the blood saving effects in patients undergoing CABG, reflected in the need for intra- and post-operative packed cell transfusion. The Caproamin Fides is also associated with lower post-operative clinical outcomes and it did not adversely affect graft thrombosis after CABG. Furthermore, it results in a shorter duration of hospitalization.

## Ethical issues

The study was approval by the Research Ethics Committee of Iran University of Medical Sciences.

## Competing interests

Authors declare no conflict of interest in this study.
